# Anodal block permits directional vagus nerve stimulation

**DOI:** 10.1038/s41598-020-66332-y

**Published:** 2020-06-08

**Authors:** Umair Ahmed, Yao-Chuan Chang, Marina Cracchiolo, Maria F. Lopez, Jacquelyn N. Tomaio, Timir Datta-Chaudhuri, Theodoros P. Zanos, Loren Rieth, Yousef Al-Abed, Stavros Zanos

**Affiliations:** 1 Institute of Bioelectronic Medicine, Feinstein Institutes for Medical Research, Manhasset, NY 11030 USA; 2 Biorobotics Institute, Scuola Superiore Sant’Anna, Pisa, Italy

**Keywords:** Action potential generation, Autonomic nervous system, Electrophysiology, Electrophysiology, Neurophysiology, Preclinical research, Biomedical engineering

## Abstract

Vagus nerve stimulation (VNS) is a bioelectronic therapy for disorders of the brain and peripheral organs, and a tool to study the physiology of autonomic circuits. Selective activation of afferent or efferent vagal fibers can maximize efficacy and minimize off-target effects of VNS. Anodal block (ABL) has been used to achieve directional fiber activation in nerve stimulation. However, evidence for directional VNS with ABL has been scarce and inconsistent, and it is unknown whether ABL permits directional fiber activation with respect to functional effects of VNS. Through a series of vagotomies, we established physiological markers for afferent and efferent fiber activation by VNS: stimulus-elicited change in breathing rate (ΔBR) and heart rate (ΔHR), respectively. Bipolar VNS trains of both polarities elicited mixed ΔHR and ΔBR responses. Cathode cephalad polarity caused an afferent pattern of responses (relatively stronger ΔBR) whereas cathode caudad caused an efferent pattern (stronger ΔHR). Additionally, left VNS elicited a greater afferent and right VNS a greater efferent response. By analyzing stimulus-evoked compound nerve potentials, we confirmed that such polarity differences in functional responses to VNS can be explained by ABL of A- and B-fiber activation. We conclude that ABL is a mechanism that can be leveraged for directional VNS.

## Introduction

Electrical stimulation of peripheral autonomic nerves has the potential to treat conditions in which the nervous system is affected or implicated. For example, vagus nerve stimulation (VNS) has been approved for certain forms of epilepsy^1,2^ and depression^3^ and is being tested in the treatment of heart failure^4,5^, rheumatoid arthritis^6^, lupus^7^, inflammatory bowel disease^8^, pulmonary hypertension^9^, arrhythmias^10^, neurorehabilitation^11^, etc. The cervical vagus is an important clinical target for invasive neuromodulation, as it is easily accessible surgically and requires a minor operation for electrode implantation^12,13^. It is also a powerful and practical physiological tool to study the peripheral and central neural circuits in which the vagus is involved, in such diverse autonomic functions as immunomodulation^14^, metabolism^15^, gut-brain axis^16^, neural regulation of cardiac function^17^ and breathing^18^, among others. At the cervical level, the vagus contains several fiber types, of different sizes (small to large), myelination patterns (unmyelinated and myelinated), and directions (afferent or efferent), mediating different functions in different organs^19^; more specifically it contains: A-type, afferent and efferent, B-type, mostly efferent, and C-type, mostly afferent, fibers. The fiber populations engaged by VNS determine its therapeutic effects, as well as undesirable off-target effects that may limit therapeutic efficacy. For these reasons, selective fiber in VNS is desirable^20^.

One way to increase selectivity of nerve stimulation is directional biasing, i.e. preferential activation of afferent or efferent fibers, depending on the intended therapeutic effect, using the mechanism of anodal block^21–23^. Anodal block can in principle selectively bias activation towards afferent (A-type) or efferent (B-type) fibers with square waveforms^21–23^. Smaller, high-threshold C-type fibers are harder to engage with square waveforms, unless high stimulus intensities are used^22,24^. Bipolar nerve stimulation causes depolarization and generation of action potentials near the cathode. When the elicited action potentials reach sufficiently hyperpolarized sections of axon near the anode, conduction of some of them is blocked. In this way, cathode caudad polarity favors the activation of efferent fibers, whereas cathode cephalad favors the activation of afferent fibers. VNS therapies have made use of this biasing mechanism: cathode cephalad polarity is used in the treatment of epilepsy^1,2^, where the desired effect is predominantly afferent, and cathode caudad polarity has been used in the clinical testing of VNS in heart failure^4,5^, or other potential applications^10,25^, where the desired effect is predominantly efferent. Despite that, functional evidence for directional biasing of fiber activation using anodal block is rare and inconclusive: it was successfully shown in some studies^21,23^, but not in others^26,27^. It has been shown that VNS polarity has an effect on stimulus-evoked fiber activation^23^, but there were no corresponding functional or physiologic outcomes reported; similarly^28^ an effect of polarity on an efferent index of activation has been reported, but without documentation of afferent effects or of evoked activation of nerve fibers. It is therefore unclear whether anodal block in VNS is a viable mechanism to directionally control fiber activation in VNS and, if so, what degree of directional selectivity it can provide.

In this study, we conducted a series of vagotomy experiments to establish physiological markers of afferent and efferent vagal fiber activation in response to cervical VNS: stimulus-elicited changes in breathing rate and heart rate, respectively. We delivered bipolar VNS with both polarities, using stimulation parameters that activate fibers of different sizes, and both afferent and efferent directionality. We found that, for a range of stimulation parameters, cathode caudad polarity was associated with a more efferent pattern of physiological responses, and cathode cephalad with an afferent pattern. At the same time, stimuli with cathode cephalad polarity blocked activation of A- and B-type fibers in the efferent direction in a current intensity-, pulse width- and distance-dependent manner, whereas stimuli with cathode caudad polarity did not, suggesting block of nerve conduction at the caudal anode. Our study provides concrete physiological and neurophysiological evidence that anodal block is a viable mechanism for functionally demonstrable directional biasing in VNS, for a range of clinically relevant stimulation parameters.

## Results

### Stimulus-elicited changes in breathing rate indicate afferent vagal fiber activation and those in heart rate indicate efferent activation

**Figure 1:**
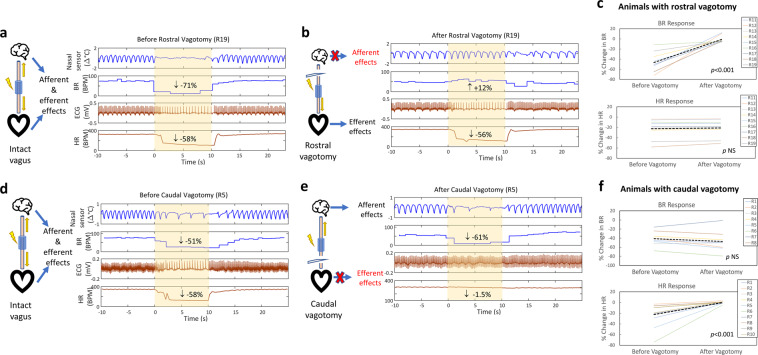
Acute physiological markers of afferent and efferent vagal fiber activation. (**a**) Acute physiological effects of cervical VNS (intensity 200 μA, pulse width 1000 μs, pulsing frequency 30 Hz) in an animal (R19) with intact vagus. Decreases in breathing rate (BR, −71%) and heart rate (HR, −58%) are observed during VNS. (**b**) After rostral vagotomy in that animal, the effect of VNS with the same parameters on BR disappears (+12%), whereas effects on HR remains intact (−56%). (**c**) The same trend for both BR and HR responses to VNS were observed in 9 animals subjected to rostral vagotomy (paired *t*-test, *p* < 0.001 top panel; *p* NS bottom). (**d**) BR and HR responses to cervical VNS (intensity 100 μA, pulse width 1000 μs, frequency 30 Hz) in another animal (R5) with intact vagus. (**e**) After caudal vagotomy, the HR response disappears, whereas the BR response remains intact. (**f**) Similar trends for BR and HR responses to VNS observed in 8 animals subjected to caudal vagotomy (paired *t*-test, *p* NS top panel; *p* < 0.001 bottom).

In order to determine physiological indicators of afferent and efferent vagal fiber activation in response to cervical VNS, experiments were performed in which both polarity configurations of VNS (cathode cephalad and cathode caudad) were first delivered with the vagus intact, and, using the same stimulation parameters, after rostral or caudal vagotomy; the configuration with the greater response before the vagotomy was included in the analysis. Physiological effects that vanished after a rostral vagotomy were deemed to be indicative of afferent fiber activation, whereas effects that vanished after a caudal vagotomy were considered indicative of efferent fiber activation. In animals with an intact vagus, cervical VNS at supra-threshold intensity produced a drop in both heart rate (HR) and breathing rate (BR) (Fig. 1a); after rostral vagotomy, the drop in breathing rate disappeared but there was still a comparable drop in HR (Fig. 1b). In 9 animals that eventually underwent rostral vagotomy (group 1), there was an average reduction of 22.3% in HR and 47% in BR with intact vagus; after the vagotomy, the HR response was comparable to that in the intact vagus experiment (−21.7%) whereas the BR response disappeared (−0.7%) (Fig. 1c; Paired *t*-test, *p* < 0.001 top panel; *p* NS bottom). This indicates that VNS-elicited change in BR (∆BR) is an index of afferent fiber activation. In a second group of animals the BR response with intact vagus (Fig. 1d) remained after caudal vagotomy (Fig. 1e), whereas the HR response with intact vagus (Fig. 1d) disappeared after the vagotomy (Fig. 1e). In 10 animals that eventually underwent caudal vagotomy (group 2), there was an average change of −22.4% in HR and −39.5% in BR; after the vagotomy, the BR response remained (−46%), whereas the HR response disappeared (−0.05%) (Fig. 1f; Paired *t*-test, *p* NS top panel; *p* < 0.001 bottom). This indicates that VNS-elicited change in heart rate (∆HR) is an index of efferent fiber activation. Stimulus polarity did not affect which afferent and efferent responses survived or disappeared after vagotomy. Additionally, no differences were observed between the left and right vagotomies (data not shown).

### Cathode cephalad polarity elicits a greater breathing rate response, whereas cathode caudad a greater heart rate response

**Figure 2:**
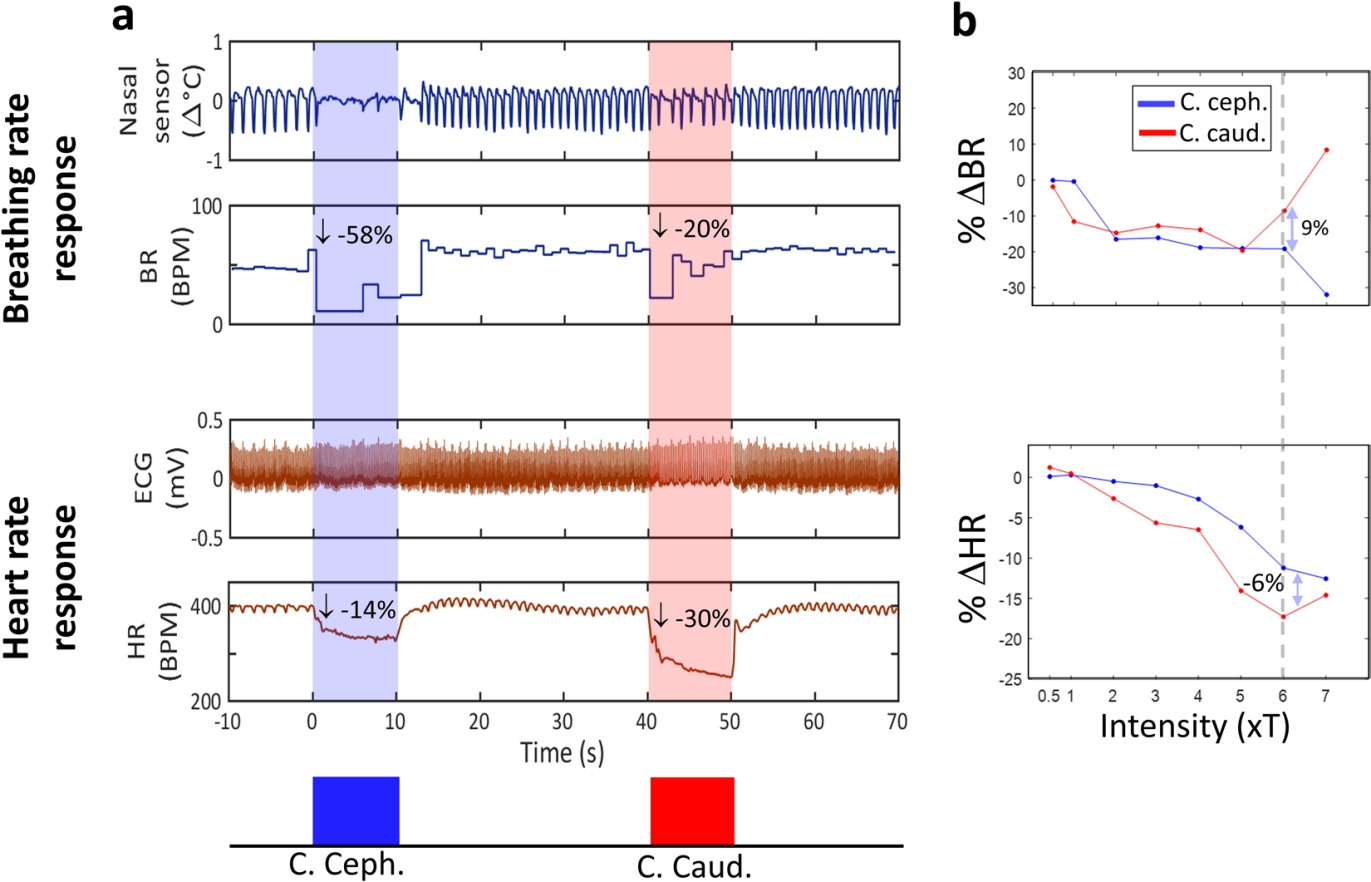
Afferent and efferent physiological responses to VNS of opposite polarities. (**a**) Afferent (breathing rate, BR) and efferent (heart rate, HR) responses to individual VNS trains of stimuli of 2 opposite polarities: cathode cephalad (blue trace), followed by cathode caudad (red trace). Cathode cephalad polarity was associated with a stronger afferent response (−58% change in BR and −14% change in HR), whereas cathode caudad with a stronger efferent response (−20% change in BR and −30% in HR). Stimulation parameters for both trains: 200 μA, 500 μs, 10 s-long train (300 pulses), 30 Hz. (**b**) Example of BR responses (top panel) and HR responses (bottom panel) to VNS of 2 opposite polarities, at a range of current intensities. Current intensity was normalized with respect to “physiological threshold” (T). In this case, cathode caudad polarity typically elicited greater drops in BR, whereas cathode cephalad greater drops in HR. Each of the 2 two-headed arrows denotes the difference in the corresponding physiological response between the 2 polarities at an intensity level of 6xT: response to cathode caudad minus response to cathode cephalad.

Efferent and afferent physiological responses (∆HR and ∆BR, respectively) were registered with VNS trains, first delivered with one polarity (cathode caudad or cathode cephalad, randomly chosen) and then with the opposite polarity. The average threshold for eliciting such responses was 145.3 ± 86.8 μA (right: n = 10 animals, left: n = 7). The threshold difference between right and left VNS was not significant. At threshold level, average ∆BR was 19–25%, and ∆HR 2–4% (Table 1). Stimuli with cathode caudad polarity elicited a greater drop in HR, whereas stimuli with cathode cephalad polarity elicited a greater drop in BR, compared to the opposite polarity (Fig. 2a), for a range of stimulus intensities (Fig. 2b). Overall, a similar trend was observed for stimulus intensities at and above physiological threshold (Table 1) and for short and long pulse widths (Table 2). A 3-way analysis of variance revealed that polarity was a significant factor for both ∆BR and ∆HR (p = 0.014 and p < 0.01, respectively), as were intensity and pulse width (p < 0.01 for both factors, for both responses). Between-polarity difference in the physiological responses compared to the magnitude of the physiological responses themselves was not trivial: 5–35% of the BR responses (e.g. for suprathreshold-intensities, between-polarity difference in the BR responses was 3%, when the responses themselves for the 2 polarities were −24 and −21%), and 30–50% of the HR responses. Higher intensity was associated with greater physiological responses, and also greater between-polarity differences in ∆HR but not in ∆BR (Table 1). Longer pulse width was associated with significantly greater physiological response but similar, in absolute terms, polarity differences for both ∆HR and ∆BR (Table 2). This indicate that cathode cephalad polarity produces a greater afferent and a smaller efferent physiological response. Additionally, BR and HR responses were significantly different between left and right VNS (Table 3). With left VNS, average ∆BR was 37–44%, and ∆HR was 4–5%; with right VNS, ∆BR was 25–28%, and ∆HR was 6–10%. Between-polarity differences in the BR response were 20% and 10% for left and right VNS, respectively. Between-polarity differences in the HR response were 13% and 65% for left and right VNS, respectively. These results indicate that left VNS is associated with a greater afferent response and a greater “gain” in the magnitude of the afferent response with cathode cephalad polarity; conversely, right VNS is associated with a greater efferent response and a greater gain in the magnitude of the efferent response with cathode caudad polarity.

**Table 1:** Mean (±SEM) afferent (percent change in breathing rate, ∆BR) and efferent (percent change in heart rate, ∆HR) responses to the 2 polarities, and mean pair-wise polarity difference (cathode caudad - cathode cephalad), for trains of stimuli with intensities at and above physiological threshold.

	Threshold-intensity (122 trains)			Supra-threshold intensity (75 trains)		
	C. caud.	C. ceph.	Pol. diff.	C. caud.	C. ceph.	Pol. diff.
∆BR (%)	−19.4 ± 3.42	−25.1 ± 3.30	5.75 ± 2.12*	−21.20 ± 4.73	−24.23 ± 4.86	3.03 ± 2.97
∆HR (%)	−4 ± 0.67	−2.5 ± 0.37	−1.5 ± 0.59* **	−13.7 ± 1.29	−9.7 ± 1.13	−4 ± 1.10* **

A single asterisk next to a polarity difference value denotes statistically-significant difference between the 2 polarities, for that physiological response (paired t-test p < 0.01); a double asterisk denotes statistically-significant difference between threshold and supra-threshold intensities, for that physiological response (2-sample t-test, p < 0.01).

**Table 2:** Similar to Table 1, but this time for trains of stimuli with short pulse widths, between 60 and 100 μs, and with long pulse widths, above 240 μs.

	Short Pulse widths (221 trains)			Long Pulse widths (243 trains)		
	C. caud.	C. ceph.	Pol. diff.	C. caud.	C. ceph.	Pol. diff.
∆BR (%)	−16.2 ± 2.88	−22.9 ± 2.5	6.7 ± 2.11*	−39.6 ± 2.9	−42.1 ± 2.94	2.5 ± 1.75
∆HR (%)	−5.7 ± 0.55	−3.4 ± 0.34	−2.3 ± 0.46*	−8.7 ± 0.54	−6.3 ± 0.41	−2.4 ± 0.46*

Responses observed with pulse widths 100 μs or shorter were similar to each other; responses with pulse widths 240 μs or longer were similar to each other. Therefore, pulse widths were categorized in one of 2 groups: either short (<=100 μs) or long (>= 240 μs).

**Table 3:** Similar to Table 1, but this time for trains of stimuli with left VNS, and right VNS.

	Left VNS (212 trains)			Right VNS (304 trains)		
	C. caud.	C. ceph.	Pol. diff.	C. caud.	C. ceph.	Pol. diff.
∆BR (%)	−37.3 ± 2.95	−44.4 ± 3.12	7.12 ± 1.64* **	−28.2 ± 2.51	−25.59 ± 2.24	−2.65 ± 1.57 **
∆HR (%)	−4.90 ± 0.49	−4.34 ± 0.38	−0.56 ± 0.50 **	−9.89 ± 0.58	−5.96 ± 0.42	−3.92 ± 0.45* **

### Cathode cephalad polarity elicits more frequently an afferent net vagal response than cathode caudad polarity

**Figure 3:**
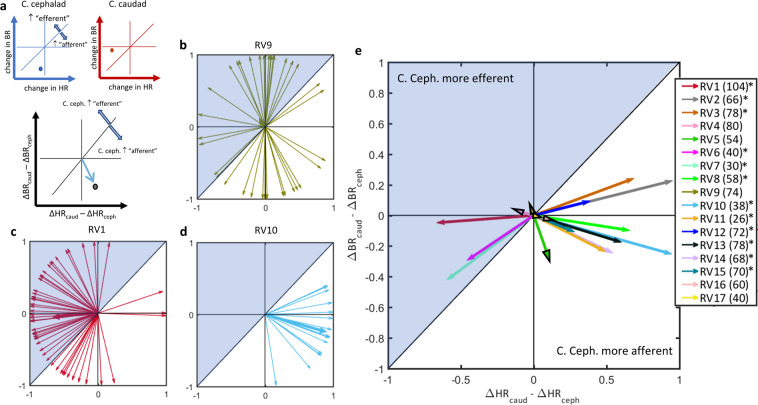
Net vagal responses to trains of stimuli of opposite polarities. (**a**) With cathode cephalad polarity (top left, blue axis), afferent (BR) and efferent (HR) responses are registered, represented as a point in the 2-D plot; horizontal and vertical lines denote zero change in BR and in HR, respectively. Points below unity line represent response patterns that are more afferent than efferent. With cathode caudad polarity (top right, red axis), the point lies above unity line, suggesting a greater efferent effect. By subtracting the afferent and efferent responses associated with each polarity (cathode caudad minus cathode cephalad), a “polarity-difference” vector plot was obtained (bottom panel). Vectors that point downwards the unity line represent stimuli for which the cathode cephalad polarity has an “afferent” net vagal response, and vice-versa for vectors pointing upwards. (**b**) Polarity difference vectors computed from 74 VNS trains in one animal (RV9). Vector directions span the entire angle space, suggesting no overall effect of polarity on the net vagal responses. Consistent with that, the vector sum (thicker vector) is short in length and the distribution of vector directions was uniform (Rayleigh test, *p* NS). (**c**) Polarity difference vectors in another animal (RV1, 104 VNS trains). The vector sum points upwards the unity line and the distribution of vector directions is not uniform (Rayleigh test, *p* < 0.01), indicating that cathode cephalad polarity had a significantly “efferent” net vagal response. (**d**) Polarity difference vectors in a third animal (RV10, 38 VNS trains), with vector sum pointing downwards (Rayleigh test, *p* < 0.01), indicating that cathode cephalad polarity had a significantly “afferent” net vagal response. (**e**) Preferred directions of polarity difference vectors in 17 animals (numbers of VNS trains indicated in the legend). Vector sums with black arrowheads denote animals in which the distribution of vectors was uniform (Rayleigh test *p* > 0.05). Of the 17 animals, 5 had no polarity difference, 9 had a more “afferent” and 3 had a more “efferent” net vagal response to cathode cephalad polarity (asterisks on legends indicates *p* < 0.01, Rayleigh test).

In order to determine whether stimulus polarity affects the net vagal response, regarded as a “mix” of both afferent and efferent effects, rather than afferent or efferent responses regarded separately, we quantified physiological responses to single VNS trains as a pattern (Fig. 3). We calculated “polarity difference vectors” of individual trains (Fig. 3a). The preferred direction of all vectors from a given animal and its relation to the unity line, represents the effect of polarity on the net vagal response. There were animals for which there was no clear preferred direction in the polarity difference vectors (Fig. 3b, Rayleigh test, *p* NS), animals in which cathode cephalad had a more efferent effect (Fig. 3c, Rayleigh test *p* < 0.01), and finally animals in which cathode cephalad had a more afferent effect (Fig. 3d, Rayleigh test *p* < 0.01). Overall, out of 17 animals, in 5 there was no preferred direction in the polarity difference vector sums, i.e. the 2 polarities had no difference in their net vagal effect, in 3 animals cathode cephalad had a more efferent net effect, and in 9 animals cathode cephalad had a more afferent net effect (Fig. 3e).

### Physiological effects of stimulus polarity are due to anodal block of evoked nerve compound action potentials

In order to examine whether anodal block can explain the different net vagal responses between the 2 polarities, we registered compound nerve action potentials evoked by pulses of both polarities, with different pulse widths and intensities, using a recording electrode caudal to the stimulating electrode, and extracted the magnitude of activation of different fiber types. Efferent B-fibers are thought to partially mediate the HR response to VNS^29^, whereas afferent A- and C- fibers both contribute to the BR response to VNS^30^. Given the placement of the recording electrode, a drop in fiber activation magnitude with increasing intensity in the cathode cephalad polarity would be consistent with anodal block, with the caudal stimulating electrode acting as anode.

**Figure 4:**
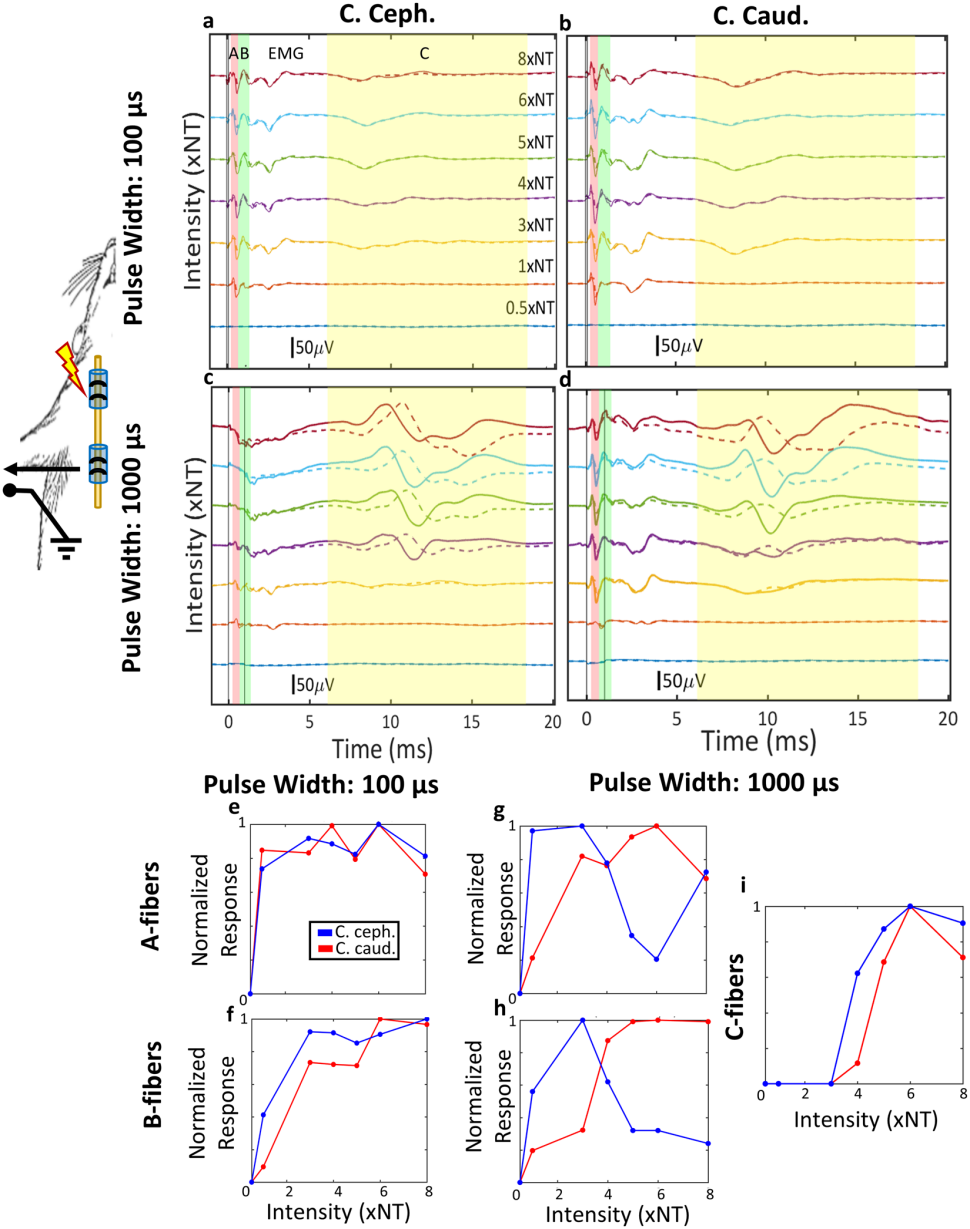
Example of the effect of stimulus polarity on A-, B- and C-fiber activation. In these experiments, VNS was delivered through a cuff placed rostrally on the cervical vagus, and stimulus-evoked compound nerve action potentials (sCNAPs) were recorded through a second cuff placed caudally (schematic diagram). (**a**) sCNAPs elicited by short pulse width (100 μs) stimuli with cathode cephalad polarity, in order of increasing intensity from bottom to top. Stimulus intensity is expressed in units of neural threshold (NT): intensity of 1 corresponds to the minimum intensity eliciting a visible neural response. Vertical shaded areas denote latency windows associated with activation of different fiber types, as explained in Fig. 1. (**b**) sCNAPs elicited by short pulse width stimuli with cathode caudad polarity. (**c,d**) sCNAPs elicited by long pulse width (1000 μs) stimuli with cathode cephalad polarity (**e**) and cathode caudad polarity (**f**), in order of increasing intensity from bottom to top. (**e,f**) Magnitude of A- and B-fiber activation, respectively, as a function of intensity of short pulse width stimuli, compiled from sCNAPs shown in panels (a,b). C-fibers were not activated at this pulse width, hence not reported here. (**g–i**) Magnitude of A-, B- and C-fiber activation, respectively, as a function of intensity of long pulse width stimuli (data from panels c,d).

Visible stimulus-evoked compound nerve action potentials (sCNAPs) were elicited at intensities of 20 ± 6.23 μA and 10 ± 3.70 μA, for 100 μs and 1000 μs pulse widths, respectively. When pulse width was short (100 μs), stimulus-evoked for the 2 polarities were similar (Fig. 4a,b). A- and B-type fiber response magnitudes were comparable across different current intensities and both A- and B-type fiber responses increased with increasing intensity, for both polarities (Fig. 4e,f). At short pulse width (100 µs), no C-type fiber activity was observed. At a longer pulse width of 1000 μs, there were differences in sCNAPs between the 2 polarities: with cathode caudad polarity (Fig. 4d), both A- and B-type fiber activity increased with increasing intensity (Fig. 4g,h), whereas with cathode cephalad polarity (Fig. 4c), A- and B-type fiber activity dropped after a certain level of intensity, indicating anodal block (Fig. 4g,h). Evoked C-type fiber activity was similar with both polarities (Fig. 4i).

**Figure 5:**
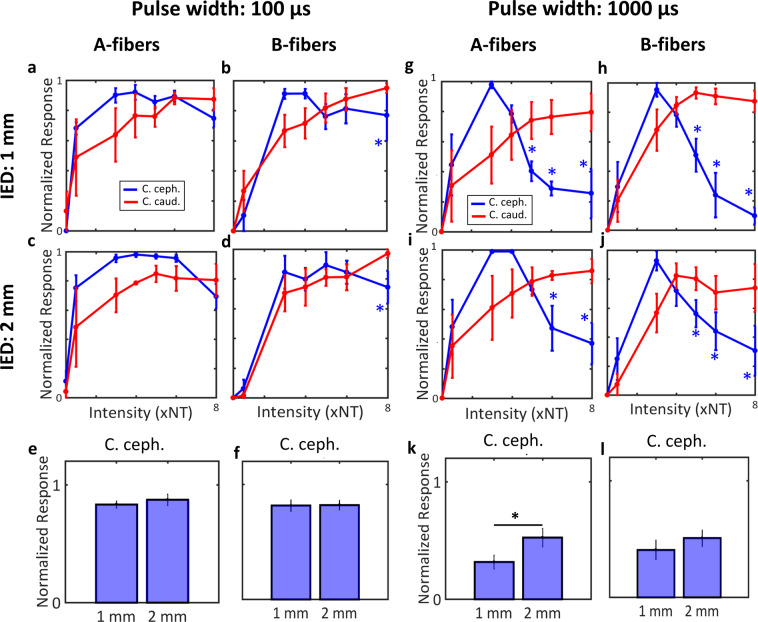
Stimulus polarity and average A- and B-fiber response magnitudes, for different pulse widths and inter-electrode distances. (**a,b**) Average (mean ±SEM, 4 animals) response magnitudes of A- and B-fibers, respectively, to stimuli with short pulse width (100 μs), delivered through a bipolar electrode with 1 mm-inter-electrode distance (IED). Shown are the responses to both polarities, as a function of stimulus intensity. Blue asterisks denote statistically significant intensity levels at which anodal block of the corresponding fibers occurred (i.e. the response to cathode cephalad polarity became smaller that of the cathode caudad; paired *t*-test, *p* < 0.05). (**c,d**) Same as before (panels a,b), with an IED of 2 mm. (**e,f**) Comparison of average response magnitudes of A- and B-fibers, respectively, for cathode cephalad polarity, between 1 mm- and 2 mm-IED, for short pulse widht: 100 μs (paired *t*-test, *p* NS). (**g,h**) Average response magnitudes of A- and B-fibers, respectively, to stimuli with long pulse width (1000 μs), delivered with an IED of 1 mm (paired *t*-test; blue asterisks denote *p* < 0.05). (**i,j**) Same as before (panels g,h), but with an IED of 2 mm (paired *t*-test; blue asterisks denote *p* < 0.05). (**k,l**) Comparison between 1 mm- and 2 mm-IED similarly to panels e and f, but for long pulse width: 1000 μs (paired *t*-test, fig. k *p* = 0.05; fig. l *p* NS).

In 4 animals, for a 100 μs pulse width, there was no evidence for anodal block in either A or B fibers, as across all intensities there was no significant difference in fiber activation magnitude between the 2 polarities (paired *t*-test, *p* > 0.05). This was true both when the inter-electrode distance (IED) of the stimulating electrodes was 1 mm (Fig. 5a,b), and when the IED was 2 mm (Fig. 5c,d). In the same animals, for a 1000 μs pulse width, A- and B-type fiber activation magnitudes were lower in the cathode cephalad polarity than cathode caudad polarity, for both 1 mm (Fig. 5g,h) and 2 mm IED (Fig. 5I,j), indicating block of nerve conduction at the anode (asterisks indicates *p* < 0.05, paired *t*-test). There was an inverse U-shaped relationship between fiber activation magnitude and current intensity for that polarity, whereas in the cathode caudad polarity that relationship was largely linear (Fig. 5g–j), indicating anodal block in the cathode cephalad polarity. For 1000 μs pulse width, the fiber activation magnitude in the cathode cephalad polarity was greater in IED of 2 mm than in IED of 1 mm, for A-fibers (Fig. 5k, Paired *t*-test, *p* = 0.05) but not significant for B-fibers (Fig. 5l, paired *t*-test, *p* = 0.2041). Finally, for 100 μs pulse width, no difference was observed between IED of 1 mm and 2 mm for both A-fibers and B-fibers (Fig. 5e,f; paired *t*-test, *p* NS).

## Discussion

The distribution of nerve fiber types in the cervical vagus nerve defines the neural substrate on which VNS exerts its actions. Selectively activating afferent or efferent fibers could significantly increase the therapeutic index of VNS, i.e. the level of desired effects for a given level of tolerable, off-target effects. Anodal block is a simple mechanism for direction-selective nerve stimulation and has been used experimentally^21–23,31–35^ and clinically^2,36–38,4,5,39^. However, its effectiveness in VNS has been inconsistent in the literature and whether it can provide functional directional engagement of vagal fibers and, if so, to what extent and under what conditions, is unknown. Although stimulation was delivered using bipolar electrodes in this study, studies have shown that anodal block can also be attained with tripolar electrodes, which may additionally minimize current leakage during stimulation^21,23,40,41^.

In the context of VNS, there are no established methods to assess functional engagement for afferent or efferent fibers, even though a method for calibrating vagal fiber type thresholds has been proposed^42^. In our study, we documented that a stimulus-elicited drop in heart rate (HR) was indicative of efferent vagal fiber activation, since it disappeared after caudal cervical vagotomy (Fig. 1d,e), and a stimulus-elicited drop in breathing rate (BR) was indicative of afferent fiber activation, as it disappeared after rostral cervical vagotomy (Fig. 1a,b). Efferent vagal, B-type, fibers ultimately affect pacemaker cells in the sinoatrial node^39,43^ and their electrical activation results in dose-dependent drop in heart rate^29^*;* severing those fibers by caudal vagotomy led to disappearance of the heart rate response. Additionally, afferent, C-type, vagal fibers have also been shown to affect heart rate, possibly through the activation of efferent fibers on the contralateral vagus nerve^33,44^. In our experiments, C-type fibers were likely not engaged, as we used intensities close to threshold or slightly above it. The exception was 1 animal in which the stimulus-elicited drop-in heart rate remained after caudal left cervical vagotomy and disappeared only after contralateral (right) cervical vagotomy (Fig. S1). On the other hand, afferent vagal, A-type and C-type, sensory fibers innervate lung stretch receptors and nociceptors, respectively, and, when stimulated, elicit various changes in breathing rate and pattern^30,45^. Similar changes in cardiac and pulmonary functional parameters in response to cervical VNS have been reported in experimental animals^27,46^ and in humans^47^, but this is the first time, to our knowledge, that these responses have been evaluated in the context of vagotomies, providing direct functional evidence for 2 easily obtainable, in experimental animals and in humans, markers of activation by VNS of efferent and afferent vagal fibers, respectively^47^.

In our study, we found that the 2 polarities differed with respect to inducing afferent and efferent physiological responses, regarded either separately (Table 1 and Fig. 2) or together, as an afferent-efferent net vagal response (Fig. 3): cathode caudad had a more efferent and cathode cephalad a more afferent functional effect, confirming the choice of polarities in clinical studies of VNS in epilepsy^1,2^ and heart failure^4,5^. Our findings are consistent with those of Ardell *et al*.^28^, in that cathode caudad produced a greater drop in heart rate than cathode cephalad polarity. Importantly, we additionally showed that cathode caudad polarity produced a more efferent net vagal response (Figs. 2a and 4), thereby ruling out the possibility that the cathode caudad polarity simply produced a more pronounced overall response than the opposite polarity, with no inherent “directionality”. We observed that higher stimulus intensities and longer pulse widths were associated with greater HR and BR responses, even though not always significantly (Tables 1 and 2). Between-polarity difference in HR responses was greater in suprathreshold, compared to threshold-level, intensities (Tables 1 and 2). In contrast, between-polarity difference in BR responses was smaller at higher intensities and longer pulse widths (Tables 1 and 2). This might be due to the activation, by stronger stimuli, of smaller diameter, higher threshold afferent C fibers, in addition to afferent A fibers; it is known that A and C fibers have different, and sometimes opposite, effects on breathing^30^. These results are consistent with the effects of pulse width and intensity in the phenomenon of anodal block of evoked volleys^21,23^. Importantly, we found that left VNS was associated with greater ‘afferent’ activation (BR response), and right VNS was associated with greater ‘efferent’ activation (HR response) (Table 3). Similarly, between-polarity difference in BR response was greater with left VNS, whereas between polarity difference in HR response was greater with right VNS (Table 3). These results provide additional evidence for selecting left VNS when afferent fibers are generally targeted (e.g. in epilepsy and depression)^1,3,36,48^, and right VNS when efferent fibers are generally targeted (e.g. in heart failure and inflammatory conditions)^4,5,7,49,50^.

In a small number of animals (3 out of 17), cathode caudad polarity had, on average, a more “afferent” net vagal response than cathode cephalad, a seemingly paradoxical effect (Fig. 3e). Such a paradoxical effect of polarity can be explained if activation of afferent fibers leads to a greater drop-in heart rate than that of efferent fibers. This could be the case if VNS activated the afferent, cardio-inhibitory aortic depressor nerve^51,52^, to a greater degree than it activated the efferent cardioinhibitory vagal fibers. Indeed, in a subset of the species of rats used in our study, the aortic depressor nerve runs as a fascicle within the cervical vagal sheath^53,54^ and could easily be activated by an all-around cuff, such as the one used in our study.

Finally, we wanted to establish that the asymmetries we documented in the physiological responses to VNS trains of opposite polarities are explainable by the mechanism of anodal block, by recording stimulus-evoked compound nerve action potentials (sCNAPs) to single pulses of both polarities (Fig. 4). Short pulse width stimuli evoked similar sCNAPs (Fig. 4a vs.4b), and comparable A- and B-fiber magnitudes (Fig. 4e,f) in both polarities, for the full range of intensities. For longer pulse width stimuli, a reduction of the size of sCNAPs with increasing current intensity in the cathode cephalad, but not in the cathode caudad polarity was observed (Fig. 4c vs. 4d), reflected in corresponding differences in the A- and B-fiber magnitudes (Fig. 4g,h, respectively). As expected, evoked C-fiber components were similar for both polarities (Fig. 4i), indicating that anodal block did not affect those high threshold, small size fibers, in agreement with^22,24^. This effect of cathode cephalad polarity is consistent with intensity- and pulse width-dependent anodal block of A- and B-type efferent-conducting impulses by the hyperpolarizing caudal anode^21,23^. In these experiments, anodal block was observed at intensities of 5–7 times neural threshold (NT) or higher, translating to approximately 50–70 μA for 1000 μs-long pulses, somewhat lower than the physiological threshold intensities of 145.3 ± 86.8 μA for 30 Hz trains of 100 μs-long pulses. Similar effects on A- and B-fiber responses were seen when bipolar stimulation was delivered through the 2 corner contacts of a tripolar cuff, with an inter-electrode distance (IED) of 2-mm instead of 1 mm (Fig. 5c,d,i,j). Importantly, A-fiber response to cathode cephalad polarity was significantly smaller in the 1-mm than in the 2-mm IED (Fig. 5k), whereas B-fiber response was also smaller, but not significantly (Fig. 5l). This provides further support to the anodal block mechanism as, when the distance between the anode and cathode is longer, some A- and B-fiber volleys escape the anodal block.

Anodal block with rectangular waveforms has not always been successful in the literature. For example, stimulus polarity was not found to have an independent effect on cardiovascular responses to VNS^27^. Also, no evidence for anodal block was seen in studies of sCNAPs^26,55,56^. There are several potential reasons for these discrepancies. First, in the sCNAP studies, there were no functional read-outs. We found that even at short pulse widths, the 2 polarities had different afferent and efferent physiological responses (Table 2), even though the magnitudes of sCNAPs were similar (Fig. 4a–d). Relying solely on a sCNAP-based read-out might underestimate the amount of anodal block; incomplete block of impulses might not be evident in sCNAPs but might still manifest itself functionally. Second, at higher stimulus intensities, the phenomenon of anodal excitation^21,23,57^ can complicate the results; for example, anodal block can be diminished at higher intensities, presumably because anode evokes generation of impulses^23^. Most of our experiments were performed at current intensities close or moderately above threshold, hence anodal excitation was unlikely. Third, the choice of pulse width and inter-electrode distance is critical for anodal block. We found that stimulus polarity had an effect on physiological responses (Fig. 2) for both short and long pulse widths, but only for long pulse width on sCNAPs (Fig. 5). In the study by Castoro *et al*.^56^, many of the pulse widths used (0.01–1 ms) were only marginally longer than the conduction time between cathode and anode of fast A-fibers (0.04–1 ms); for slower, B-fibers, the pulse widths were even shorter than the conduction time (0.6–2.5 ms). Finally, it is important to note that despite the significant effects of polarity in our study, there was also significant variability both within and across animals. For example, in 5 out of 17 animals, physiological responses were largely symmetrical for the 2 polarities (Fig. 3e), without an obvious reason. This highlights that even in the relatively anatomically simpler rat vagus nerve a variety of responses should be expected during bipolar stimulation with rectangular pulses.

Targeted engagement of fibers in VNS could be realized using different approaches: fascicle-directed^58–62^, fiber diameter-based^20,63^ or direction-specific^21,64–67^. Histological studies show that the spatial organization of fibers in the vagus nerve is complex, as different fiber types, both afferent and efferent, are found together in single fascicles^58,68–71^. On the other hand, fibers in different fascicles might project to distinct anatomical targets, central or peripheral^58,59^. This suggests that to attain the desired degree of selectivity, combinations of several approaches might be required. Anodal block provides a significant degree of directional biasing in the vagus and it should be leveraged in a combined neurostimulation approach, as its implementation and delivery are straight-forward from an engineering perspective.

## Methods

### Animal preparation, physiologic instrumentation, surgical procedure

Forty adult male Sprague Dawley rats (age: 2–5 months, weight: 300–550 grams) were used in the study under the approval of The Institutional Animal Care and Use Committee at The Feinstein Institutes for Medical Research. All experiments were conducted in accordance with relevant guidelines and regulations. Anesthesia was induced and maintained using isoflurane (induction at 4% and maintenance at 1–2%). Adequate anesthesia level was maintained by checking pedal reflex every 15 minutes throughout the experiment. Body temperature was measured with a rectal probe and was maintained between 36.5–37.5 °C using a heating pad connected to a T-pump warm water recirculator (Stryker). Electrocardiography (ECG) was recorded by using 3 leads (intradermal needles) placed on the limbs; the positive lead was connected to left upper limb, negative lead to left lower limb and ground right lower limb, providing a single differential ECG signal. Breathing was monitored with a nasal temperature probe (T-type Thermocouple Probe, ADInstruments): nasal temperature drops during inspiration, increases during expiration. A pulse oximeter was attached on the base of the tail to measure oxygen saturation (SpO_2_ Animal Clip, ADInstruments).

**Figure 6:**
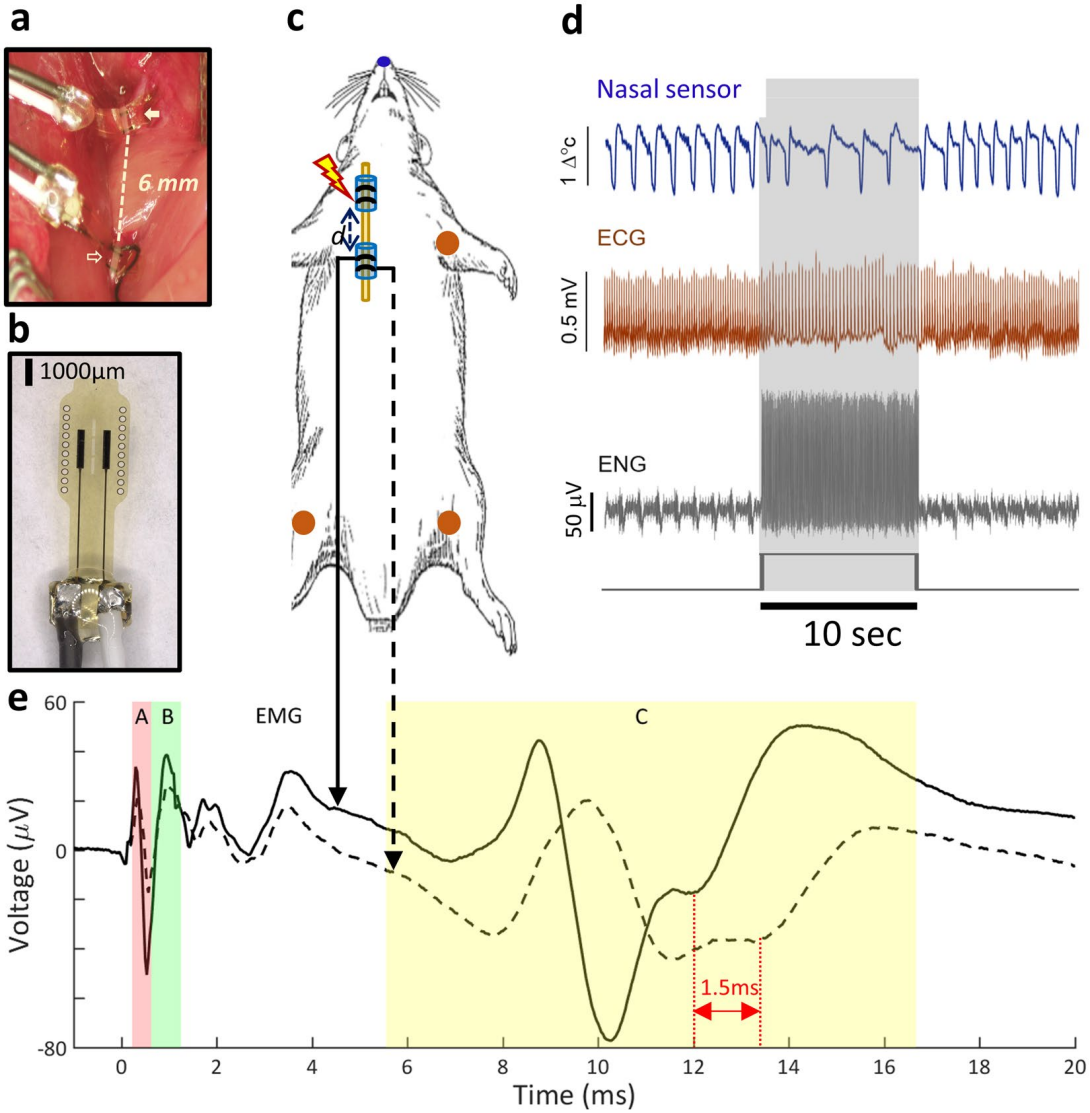
Experimental setup, electrodes, physiological sensors and signals. (**a**) Two bipolar cuff electrodes shown placed below corresponding exposed parts of the cervical vagus nerve in a rat, at a distance of 6 mm from each other. The rostral electrode (filled arrow) is used for stimulation, the caudal (open arrow) for recording. (**b**) Close-up image of a bipolar, Flex electrode. (**c**) Schematic of the animal with the 2 vagus nerve electrodes and the sensors for physiological measurements: nasal temperature sensor for resolving air flow (blue), electrocardiogram (ECG) sensors (2 differentials and 1 ground) for registering ECG (red). (**d**) Snippet of signals acquired during delivery of a 10 s-long (300 pulse count) VNS train (rectangular black trace at bottom panel): temperature signal from nasal sensor (top panel, blue trace), voltage signal from ECG sensor (second panel, red trace), electroneurogram (ENG) signal from recording nerve electrode (third panel, grey trace). In the raw ENG signal, periodic modulations associated with breathing and stimulation artifacts associated with VNS were also observed. (**e**) Stimulus-evoked compound nerve action potential (sCNAP) compiled by averaging single ENG sweeps around the onset of each of the 300 stimuli in the VNS train. The solid and the dotted trace represent the 2 sCNAPs recorded on the 2 contacts of a bipolar recording cuff; notice the time-shift in the 2 traces for slow-conducting C-fibers, corresponding to different arrival times of CNAPs at the 2 recording contacts, and the lack of time-shift for extra-neural EMG, which is recorded simultaneously on both contacts. The colored shaded areas represent the windows of latencies (minimum and maximum latency) for each of the 3 fiber type and EMG activity: red for A fibers (0.3–0.95 ms), green for B fibers (0.7–1.2 ms), yellow for C fibers (5.5–16.6 ms) and white for EMG (2–10 ms). The latency windows were calculated based on the known conduction velocities of fibers types and distance between stimulating and recording electrodes. The magnitude of activation for each fiber type was calculated as the amplitude of the CNAP component within each of the 4 latency windows.

For placement of the vagus nerve electrodes, a midline 2–3 cm incision was initially made on the neck area. Salivary glands were separated, and muscles were retracted to reach the carotid bundle. Using a dissecting microscope (Stereomicroscope SteREO Discovery.V8, Carl Zeiss Microscopy), the left (n = 22) or right cervical vagus nerve (n = 18) was isolated, first at the caudal end and then at the rostral end of the animal. The segment of the nerve between the two isolated sites, located below the omohyoid muscle, was left intact within the carotid bundle. Two custom-made, “acute Flex” electrodes were placed on caudal and rostral sites of the exposed nerve (Fig. 6a–c). Once the electrodes were placed, the distance between the two electrodes was measured and ranged between 5.5 and 6 mm across experiments. A silicone elastomer (Kwik-sil, World Precision Instruments) was laid around each of the electrodes to reduce current shunting during electrical stimulation. Once the elastomer cured, a pool of body-temperature saline was used to keep the nerve and the surrounding surgical field hydrated. Electrical stimulation was delivered to the rostral cuff, whereas the caudal cuff was used for recording nerve activity (Fig. 6c).

### Electrodes

“Acute Flex” electrodes used in this study were made using a microfabrication process. Briefly, the electrodes were fabricated on spin-cast polyimide films with sputter deposited bond-pad and trace metallizations, followed by sputter deposited iridium oxide (IrOx) electrodes sites. IrOx was chosen because it provides low impedance with stable stimulation characteristics. The exposed electrode contact sites were rectangular in shape with dimensions of 1,418 µm ×167 µm and center to center spacing between the electrodes of 1 mm. Electrode Lead wires were silicone-insulated stranded copper wires (Cooner Wire Co). Typical electrode impedances in saline ranged from 0.5 to 1 kΩ and after electrode placement in the animal ranges increased to 2 to 3 kΩ. An assembled bipolar “Flex” electrode is shown in Fig. 6b.

### Physiological and neural signal recordings, and nerve stimulation

Physiological signals were pre-amplified using a Bio-Amplifier for ECG, an Oximeter Pod for oxygen saturation, and 2 Temperature Pods for nasal temperature and rectal temperature (ADInstruments). Conditioned signals were digitized on PowerLab 16/35 (ADInstruments) at sampling rate of 1 kHz. They were then streamed to a PC running LabChart v8 (ADInstruments; Fig. 6d). Nerve activity was recorded from both contacts of the caudal cuff in a single-ended configuration, with the reference electrode on nearby salivary gland. The signal was pre-amplified and digitized at a sampling rate of 30 kHz, on an RHS2000 headstage (Intan Technologies), connected to an Intan 128 channel Stimulation/Recording controller; the controller streamed the data to a PC running a compiled version 1.05 Intan Stimulation/Recording controller software (Fig. 6d,e). Both the physiologic and the neural recording systems received common pulse-onset and train-onset sync-output signals from the neurostimulator, sampled at 30 kHz and 40 kHz, respectively. These 2 shared signals allowed synchronization of the data recorded from the 2 separate streams.

For nerve stimulation, we used the STG4008 stimulus generator (Multichannel Systems) in constant current mode, programmed using MC_Stimulus II v3.5.2 and current waveform parameters were generated by custom-made MATLAB (Mathworks) scripts. The voltage drop across the stimulating electrode was also registered on the ADI system at a sampling rate of 40 kHz; the signal buffered using a custom-made amplifier connected in parallel with the stimulating electrode, to subtract the stimulus artifact from the stimulus-evoked potentials and ensure that the parameters of stimulus pulses and trains were as intended^72^.

### Vagotomy experiments

In order to identify physiological indicators of afferent and efferent fibers, vagotomy experiments were conducted. These experiments consisted of two types of vagotomy: rostral to the stimulating electrode (rostral vagotomy) and caudal to stimulating electrode (caudal vagotomy). One stimulating electrode was used during these experiments and all physiological signals were recorded as described above. A stimulus train was initially applied to ensure vagal fiber recruitment, confirmed by a significant, at least 10%, change in heart rate (∆HR) and breathing rate (∆BR). After the initial stimulation, rostral or caudal vagotomy was performed using scissors without disturbing the stimulating electrode. Once vagotomy was performed, the stimulation paradigm was repeated. After the physiological responses were registered, the animal was euthanized.

### Vagus nerve stimulation experiments

After all sensors and electrodes were placed, physiological and neural response thresholds were determined for the stimulating electrode. The physiological threshold (T) was defined as the minimal current intensity of pulses of either polarity that elicited an approximately 5–10% drop in heart rate and/or 5–10% decrease in breathing rate (whichever was lower); the neural threshold (NT) was defined as the minimal current intensity of pulses of either polarity that elicited a visible neural response at the recording electrode. Stimulation parameters used for finding the 2 thresholds were: trains of monophasic pulses with a pulse width of either 100 μs or 1000 μs, a pulsing frequency of 30 Hz and a train duration of 10 s. Once thresholds were determined, stimulation events consisting of trains of pulses of different polarities (cathode caudad and cathode cephalad), pulse widths (60 μs, 100 μs, 240 μs, 500 μs, 1000 μs), intensities (0.5xNT, 1xNT, 3xNT, 4xNT, 5xNT, 6xNT, 8xNT and 0.5xT, 1xT, 2xT, 3xT, 4xT, 5xT, 6xT, 7xT) and frequencies (10 Hz, 30 Hz) were delivered every few minutes while registering physiological and neural responses to VNS. Each stimulus train with a given combination of parameters was delivered twice, in random order. Before delivering another stimulus train, physiological parameters (heart rate, breathing, oxygen saturation and temperature) were required to have returned to baseline levels. Only one stimulating electrode was used in experiments in which physiological responses to VNS were tested.

## Analysis

### Analysis of physiological parameters

All physiological and neural data were processed using custom-made software on MATLAB (Mathworks). For ECG signals, a high-pass filer at 0.1 Hz was first applied to eliminate direct current (DC) voltage drift, followed with a QRS peak detection to compute heart rate (HR) based on individual R-R intervals. Minimum R-wave amplitude and R-R interval values were set for each animal, respectively, to improve the detection precision and suppress false R-wave detection events as a result of the VNS artifact. The nasal sensor signal was first pre-processed by a high-pass filter at 0.1 Hz to remove the temperature drift. A 20-point moving average window was used to smooth and reduce ripple on top of signals. The breathing cycles were computed by detection of both positive and negative peaks for a precise detection of each breath.

For each stimulation train, the change in heart rate (∆HR) and in breathing rate (∆BR) were computed as follows:

$$\triangle {HR}=\frac{{{HR}}_{{pre}}-{{HR}}_{{VNS}}}{{{HR}}_{{pre}}}$$

$$\triangle {BR}=\frac{{{BR}}_{{pre}}-{{BR}}_{{VNS}}}{{{BR}}_{{pre}}}$$

where HR_pre_ (or BR_pre_) corresponds to the mean HR (or BR) during the 10 s-long epoch before the stimulus train was delivered, and HR_VNS_ (or BR_VNS_) to the mean HR (or BR) during the delivery of the stimulus train. Each ∆HR (and ∆BR) value was then normalized with respect to the maximal value computed in that animal.

### Analysis of patterns of physiological responses to VNS

The patterns of afferent and efferent physiological responses to a single VNS train and the difference between the 2 stimulation polarities (cathode caudad vs. cathode cephalad) were further analyzed using a vector approach.

We defined the “polarity difference” vector *V* for a stimulus train *j* delivered to animal *i* as:

$${V}_{i,j}=[\begin{array}{c}{\triangle {HR}}_{{caud}}-{\triangle {HR}}_{{ceph}}\\ {\triangle {BR}}_{{caud}}-{\triangle {BR}}_{{ceph}}\end{array}]$$

where ∆HR_caud_ is the normalized change in HR associated with the cathode caudad polarity and ∆HR_ceph_ was that associated with the cathode cephalad polarity (and similarly with breathing rates). The 2-D vector *V*_*i*_,_*j*_ then represents the difference in the efferent (HR) response between the 2 polarities as the vector length on the x-axis, and in the difference in the afferent (BR) response between the 2 polarities on the y-axis (Fig. 3a). For example, a vector length of 0 in the x-axis means no HR response difference between the 2 polarities, and a vector length of 0 in both axes means neither HR nor BR response difference between the 2 polarities. The sign and magnitude of differences in the HR and BR responses between the 2 polarities together define the direction of the 2-D polarity difference vector *V*, calculated for single VNS trains. Polarity difference vectors pointing downward the unity line (at an angle between 225 and 45 degrees) correspond to stimulus trains in which cathode cephalad polarity has a more afferent effect (and therefore cathode caudad polarity a more efferent effect); the opposite for vectors pointing upward the unity line (Fig. 3a).

Finally, the overall physiological effect in each animal was computed in vector form as the sum of all vectors *V* for that animal (Fig. 3b–d):

$${\bar{{\boldsymbol{V}}}}_{i}=\sum _{j}{norm}({V}_{i,j})$$

### Analysis of fiber activation magnitude

A high-pass, 3^rd^ order Butterworth filter with a corner frequency of 1 Hz was applied to the nerve recordings to remove the low frequency components of the signal. Nerve recording sweeps were extracted around each of the pulses in a stimulus train using the registered pulse sync triggers, and sCNAPs were compiled by averaging the sweeps across all pulses in a stimulus train (Fig. 6e). A stimulus artifact suppression algorithm was applied to resolve very short latency fiber responses and improve nerve signal quality^72^. Briefly, a custom-made differential amplifier was used to record the voltage drop across the pair of stimulating electrodes. The stimulation voltage drop was scaled to match, in terms of amplitude, the corresponding voltage deflection in the sCNAP and was then subtracted from the sCNAP. The magnitude of stimulus-evoked A-, B- and C-type fiber activation was computed by the peak-to-trough amplitude of the sCNAP within respective time windows determined by the conduction velocity of each fiber group and the center-to-center distance between stimulating and recoding electrodes. The fiber conduction velocities were taken to be 5–120 m/s for A fibers, 2–8 m/s for B fibers, 0.1–0.8 m/s for C fibers^19^. We have previously shown that sCNAPs include evoked EMG responses from laryngeal muscle contraction^72^; those sCNAP components, typically occurring 2–10 ms post-stimulus were excluded from our analysis. To discriminate nerve fiber activity overlapping with EMG, latencies of evoked responses on each of the 2 signals from the contacts in the bipolar recording electrode (solid and dashed traces in Fig. 6e) were compared: those latencies differed by 1–2 ms for slow-conducting nerve potentials, corresponding to the time it takes C-fiber conduction to cover 1-mm distance between the 2 contacts, whereas they were the same for extra-neural EMG signals that were recorded simultaneously on both contacts. At this inter-electrode distance, no significant latency difference was observed for the much faster A and B fibers. To compare fiber activation magnitudes across animals and different parameter combinations, all fiber responses of a given animal were normalized with respect to the maximum value measured in that animal.

### Statistical analysis

Paired *t*-test was used to compare the physiological responses before and after vagotomy. Paired t-test was used to compare physiological responses to the two stimulus polarities. Two-sample t-test was used to compare between-polarity differences between different subsets of VNS trains (threshold vs. supra-threshold intensity and short vs. long pulse width). Three-way ANOVA was used to examine the effect of polarity, intensity and pulse width on between-polarity difference. Paired t-test was used to compare the magnitudes of activation of different fiber types in response to the 2 polarities, in sCNAPs, and to compare fiber activation magnitude in response to stimuli of different pulse widths and inter-electrode distances. Rayleigh test was used to assess non-uniformity in the distribution of vectors representing the afferent and efferent physiological responses to individual trains of VNS. Comparisons were deemed statistically significant for *p* values < 0.05 for all analyses. Statistical analyses were conducted on MATLAB (Mathworks).

## Supplementary information

Supplemental information.

## Data Availability

The data that support the findings of this study are available from corresponding author upon reasonable request.
