# Supplementary material for: Anodal block permits directional vagus nerve stimulation
Source: Sci Rep. 2020 Jun 8;10:9221. doi: 10.1038/s41598-020-66332-y (PMC7280203; doi:10.1038/s41598-020-66332-y)
Supplement: Supplementary file 1 — Supplemental information. [file 41598_2020_66332_MOESM1_ESM.pdf]

# Anodal block permits directional vagus nerve stimulation

**Umair Ahmed[1]#, Yao-Chuan Chang[1]#, Marina Cracchiolo[1,2], Maria F. Lopez[1], Jacquelyn N. Tomaio[1], Timir Datta-Chaudhuri[1], Theodoros P. Zanos[1], Loren Rieth[1], Yousef Al-Abed[1], Stavros Zanos[1]*"**

[1] Institute of Bioelectronic Medicine, Feinstein Institutes for Medical Research, Manhasset NY 11030, USA

[2] Biorobotics Institute, Scuola Superiore Sant'Anna, Pisa, Italy

#: These 2 investigators contributed equally to this work

* Corresponding author: Stavros Zanos, szanos@northwell.edu

## Supplementary Figure

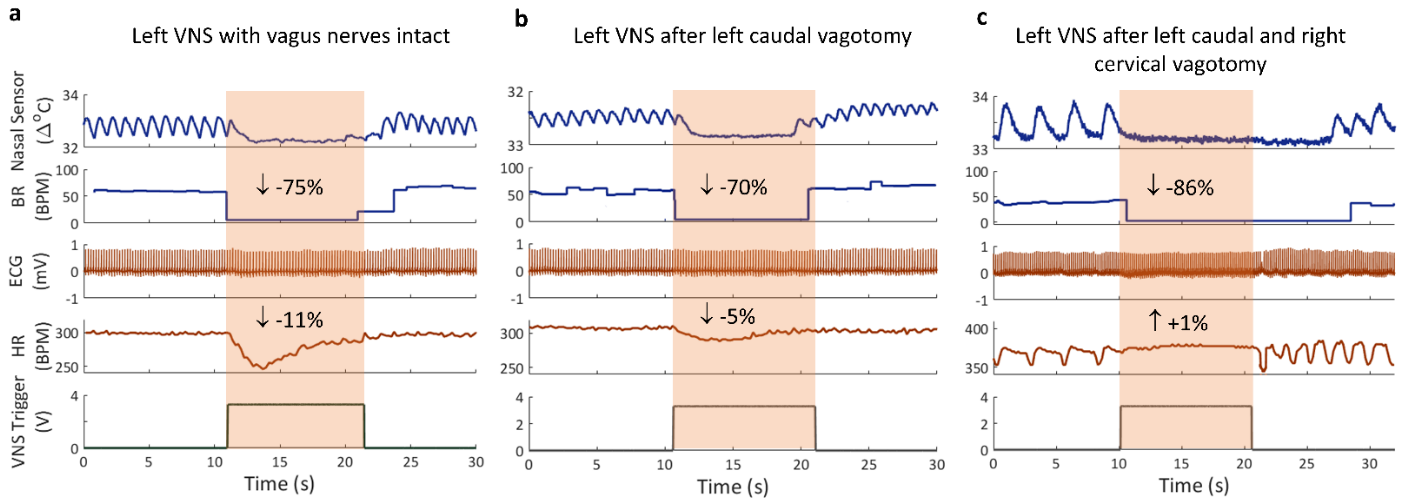


**Figure S1. Changes in breathing rate and heart rate after unilateral and bilateral vagotomy.**

a) Acute physiological effects of left cervical VNS (intensity 500 μA, pulse width 100 μs, pulsing frequency 30 Hz) with intact left and right vagus. Decreases in breathing rate (BR, -75%) and heart rate (HR, -11%) are observed during VNS.

b) After left caudal vagotomy (unilateral vagotomy) in the same animal, the effect of left VNS with the same parameters on HR diminishes (-5%), whereas effects on BR remains intact (-70%).

c) After left caudal and right cervical vagotomy (bilateral vagotomy) in the same animal, the effect of left VNS with the same parameters on HR disappears (+1%), whereas effects on BR remains intact (-86%).
